# Intussusception or Invagination

**Published:** 1893-01

**Authors:** J. F. Butterfield


### INTUSSUSCEPTION OR INVAGINATION.

By J. F. Butterfield, V.S.

Intussusception or invagination of the intestines in cattle is comparatively rare. In a practice extending over some years, in a dairy region, I have had but one case that proved to be intussusception. Most pathologists give it as the result of simultaneous distension of the investing, and spasm of the inclosed portion of the bowel, with irregular or suddenly reversed peristatic action. Its infrequency in cattle is probably due to the fact that cattle are but little subject to colics, of which it is a sequela. The cæcum is, according to Steel, frequently the seat of the lesion.

The case I wish to give you an account of occurred in my practice last winter. On February 20, 1892, Mr. H. L. Bailey, of Brooklyn, Susquehanna county, Pa., sent for me to come to his place, a distance of eight miles, to see one of a fine pair of four-year-old Devon oxen. The ox weighed about fifteen hundred pounds. On arrival, about 2 P. M., I found the animal lying down, comparatively quiet, but in a peculiar position. He lay with his hind feet more under him than is natural in health, and with a peculiar tension of the abdominal muscles.

On getting the animal on his feet he showed colicy pains, kicked up at his belly, and had that peculiar tension of the abdominal muscles as though he was trying to get some twist or knot out of his bowels. These symptoms were continuous. The temperature was normal. The pulse was slightly accelerated. The respirations were but little affected. On inquiry, I found the ox had eaten his hay and feed clean through the night. When they went to feed in the morning they found him lying down, which they knew was unusual. They got him up, but he refused to eat hay or feed, and they said he had had the symptoms I have described. He would not remain standing very long. They said he passed fæces frequently, but in small quantities, when they first found him. When I saw him no fæces passed, only a little mucus. I used warm water enemas, with no beneficial results. On exploration per rectum, I thought I detected a knot or tie of the small intestine. I attempted to reduce it by manipulation through the rectum, but did not succeed.

I then told the owner that the only hope for recovery was to perform laparotomy. The owner instructed me to do as I thought best. I accordingly secured the animal in a standing position by bringing a rope from manger along right side of ox, and secured to left hand stable partition, thereby holding him to partition, leaving right side of ox free to operate upon. Next tied hind legs together at hocks, preventing his kicking at operator. I now made an incision in right side equidistant from external angle of ilium, last rib, and transverse processes of lumbar vertebra, large enough to admit both hands, using the carbolic lotion for instruments and the bichloride solution for my hands and the wound. Introduced both hands and attempted to reduce the obstruction to the bowel, but could not find just how it was knotted. I found I could bring the obstructed portion out through opening, and did so. By the help of my eyes I was enabled to see just what the lesion was. By a little traction on the imprisoned portion I soon had it withdrawn. There was about eighteen inches of the bowel engaged in the volvulus. All of this portion was highly congested. and four or five inches extremely dark. There was what appeared to be a little hardened lump of fæces attached to the mucous coat of the intestine just where it began to unravel, which kept it from regaining its natural position by the peralstaltic action of the bowels. Next returned the gut to as natural position as possible and sutured opening in the ordinary manner.

Told the owner to keep patient quiet and not to offer feed or water until he showed an appetite for them. Had news from my patient on the third day after the operation. His bowels had moved, and he was taking feed and water with a relish. I heard again in three weeks from the owner that the ox had made a complete recovery.

Now in this case there were some points I wish to call attention to, as they may aid others in making a diagnosis:

First is the peculiar position which the ox took while lying down. The hind feet were well under the abdomen, and the abdominal muscles drawn up or a peculiar tension on them. This tension he maintained while standing also.

Second. The intestine that is intussuscepted is more or less distended with gases, which cause it to float upon the other mass of intestine, thereby elevating it in the region of the rectum, where it can be easily recognized upon examination per rectum.

Third. No elevation of temperature at first.

Fourth. Entire absence of passage of fæces after the first few hours.

It is not a very formidable operation to perform, to reduce the intussusception after one has made his diagnosis.

